# Expression variation of *OGG1* and *HPRT* gene and DNA damage in arsenic exposed industrial workers

**DOI:** 10.1371/journal.pone.0273211

**Published:** 2022-09-30

**Authors:** Zertashia Akram, Ishrat Mahjabeen, Muhammad Umair, Muhammad Fahim, Mahmood Akhter Kayani, Lulu Fatima, Malik Waqar Ahmad, Sarwat Jahan, Tayyaba Afsar, Ali Almajwal, Nawaf W. Alruwaili, Iftikhar Ali Khawar, Suhail Razak

**Affiliations:** 1 Department of Biosciences, Cancer Genetics and Epigenetics Research Group, COMSATS University Islamabad, Pakistan; 2 Faculty of Biological Sciences, Department of Animal Sciences, Quaid-i-Azam University, Islamabad, Pakistan; 3 Department of Community Health Sciences, College of Applied Medical Sciences, King Saud University, Riyadh, Saudi Arabia; 4 Division of Oncology, Department of internal Medicine, Washington University School of Medicine, St. Louis, MO, United States of America; All India Institute of Medical Sciences - Jodhpur, INDIA

## Abstract

Arsenic exposure alters redox balance, induces DNA damage, and deregulates many genes. OGG1 gene involved in base repair mechanism, for excision of 8-oxoguanine (8-oxoG) from DNA formed as a result of accumulation of ROS in cell. HPRT gene encode transferase enzymes involved in purine recycling mechanism. The main focus of the study was to evaluate the expression variation in *HPRT*, *OGG1* gene expression, and DNA damage of industrial workers. Blood samples of 300 occupational workers were collected from welding, brick kiln, furniture, pesticide, and paint industry (n = 60/industry) to evaluate the expression variation in *HPRT*, *OGG1* gene expression, and DNA damage in blood cells by comet assay along with age and gender matched 300 control individuals. Blood arsenic content was higher (P<0.001) in an industrial group compared to the control. *OGG1* and *HPRT* expression were (P<0.05) downregulated in exposed workers compared to controls. Spearman correlation analysis showed a significant positive correlation between HPRT vs OGG1 (P< 0.0001) in exposed workers compared to controls. Altered expression of both genes was observed between workers with <25years and >25years of age as well as between workers with <10years and >10year exposure. Reduced expression (P<0.05) of both genes and a high extent of DNA damage was evident in exposed smokers compared to respective non-smokers. DNA fragmentation was higher (P<0.05) in the furniture, welding and brick kiln group compared to control, and other industries. The present study suggests that altered expression of *OGG1* and *HPRT* gene induce oxidative stress, showed a negative impact on the recycling of purines leading to DNA damage which increase the vulnerability of workers to carcinogenicity.

## Introduction

Discontinuous and oscillating release of the contaminants in the environment causes organisms to be exposed to fitful, toxiferous entities [1]. Increased energy demands, agriculture, and industrialization are major initiating factors of inducing imbalance in human and environmental health [2]. This issues generally arise in third world countries, where there is improper check on hygiene, excessive emission of gases in the air, garbage discharge in water, accumulation of heavy metals in the environment from the smoke of chimneys, vehicles, dust emitted from brick kiln [3], mining activities and welding processes [4]. Heavy metals like arsenic, lead, mercury, chromium, iron, manganese, cobalt are responsible for altering the cell signaling, antioxidant balance, DNA repair mechanism, apoptosis, or inducing any mutations in genomic DNA [5].

Arsenic is a potent carcinogen and is considered a global toxic insult to human health [6]. Arsenic interacts with the environment either naturally or through anthropogenic activities. It has large scale industrial usage in the manufacture of batteries, alloyed, pigments, pesticides, furniture preservatives, coal combustion [7]. Arsenic and its metabolites may inhibit DNA repair mechanisms [8], induce oxidative stress, alter redox balance, may initiate various signal transduction pathways [9], yield increased frequency of DNA damage, and can introduce single and double strand breaks [10]. A broad range of health issues has been associated with chronic exposure to arsenic [11,12]. A positive association between high levels of urinary arsenic content and increased intensity of genetic damage was obtained in children with high exposure to arsenic. Moreover, a negative correlation was found between arsenic content and DNA repair ability in exposed children. Reduced ability of DNA repair was observed in children with high arsenic exposure compared to children with low exposure to arsenic [13]. Arsenic and its metabolites inactivate enzymes involved in the DNA repair mechanism [8]. Kiln workers and welders are at high peril of DNA damage due to occupational exposure to different genotoxic materials including arsenic present in dust, smoke [14,15], and metal fumes [16]. DNA damage in blood lymphocytes of welders was higher in tail intensity, tail moment [17], and tail length [4] compared to unexposed controls.

Oxidative stress is a major mechanism by which arsenic exerts its toxicity [18]. Upon arsenic exposure, ROS generation is the earliest response of cells [19]. ROS causes damage to cellular components such as DNA, proteins, lipids which ultimately leads to carcinogenesis [20]. The base excision repair (BER) system repairs oxidative DNA damage and it works by the participation of proteins encoded by different genes [21]. Each damaged oxidative base is removed by its specific glycosylase. The most frequent oxidative DNA damage is 8-Oxoguanine (8-OxoG) [22] which is excised by DNA glycosylase encoded by a *OGG1* gene [23]. 8-oxo-2’-deoxyguanosine is renowned reactive oxygen species and is taken as a sensitive marker of arsenic induced oxidation. In humans, the *OGG1* gene is localized on chromosome 3p25, a region frequently lost in cancer. Repairing of mutagenic base byproducts by *OGG1* is essential to maintain the integrity of the genome and to prevent cancer development [24]. Arsenic along with its metabolites inhibits the activity of enzymes encoded by *OGG1* which acts as glycosylase [25]. 8-OxoG causes transversions that might initiate activation of oncogenes or inactivation of tumor suppressor genes [26]. Exposure to environmental toxicants induces DNA damage, which results in accumulation of 8-OxoG and increasing oxidative stress would lead to genetic instability in exposed individuals. Expression of OGG1 gene acted as a biomarker of oxidative damage in occupationally exposed workers. Therefore, OGG1 expression variations would give a better baseline understanding of mechanism working behind metal induced toxicity. HPRT is used as a biomarker for the evaluation of genetic effects induced by arsenic exposure either environmental or occupational [27]. Moreover, mutations in HPRT locus are observed in a population exposed to mutagenic or carcinogenic agents for detection of DNA integrity which ultimately enhances the cancer risk. A significant association was found between increased cancer risk and HPRT mutations [28]. Significant mutations in the HPRT locus was observed in at-risk population like smokers or patients with DNA repair deficiency [28,29]. HPRT gene is member of Protein salvage pathway encode transferase enzyme used for purines recycling metabolism. Metal exposure may inhibit function of HPRT gene, and deficiency of HPRT leads to poor regulation of cell cycle and proliferation mechanisms, RNA metabolism, DNA replication and repair. Expression variation of HPRT gene would give general understanding of metal induced toxicity. Single Cell Gel Electrophoresis (SCGE) or comet assay identifies the DNA fragmentation at single-cell level [30]. The index of genetic damage was measured using different parameters of comet assay reflecting the extent of DNA damage in a single cell. So far limited data is available regarding the arsenic induced genotoxicity coupled with workers employed in different industries of Pakistan. In addition, correlation of *HPRT* and *OGG1* gene with DNA damage and metal toxicity has not explored in industrial workers. Moreover, association of genetic damage with different demographic parameters like age, exposure time, smoking status has not been explored in workers of our targeted industries. Therefore, the present study was designed to measure DNA fragmentation and to find the expression variation of *OGG1* and *HPRT* gene in arsenic exposed industrial workers and comparison with unexposed control individuals. This will be helpful for a better understanding of the mechanism acting behind arsenic induced genotoxicity.

## Results

The demographic data obtained from the control and industrial group is shown in Table 1.

**Table 1:** Demographic data of control and exposed group of furniture, paint, welding, pesticide and brick kiln industry.

Parameters	Controls	Brick kiln	Welding	Furniture	Pesticides	Paint
Sample size	300	60	60	60	60	60
Mean age	27.18±1.55^¥^	29.31±1.68^¥^	23.76±0.86^¥^	25.78±2.42^¥^	29.97±1.38^¥^	24.00±1.52^¥^
Smokers	165	29	28	33	30	28
Non-smokers	135	31	32	27	30	32
Age (<25year)	116	38	42	50	36	24
Age (≥25year)	184	22	18	10	24	26
Exposure (<10 year)	**_**	29	34	50	40	23
Exposure (≥10 year)	**_**	31	26	10	20	37

^¥^ Values expressed as Mean±SEM. Other values expressed as numbers.

### Comet assay description

**Fig 1:**
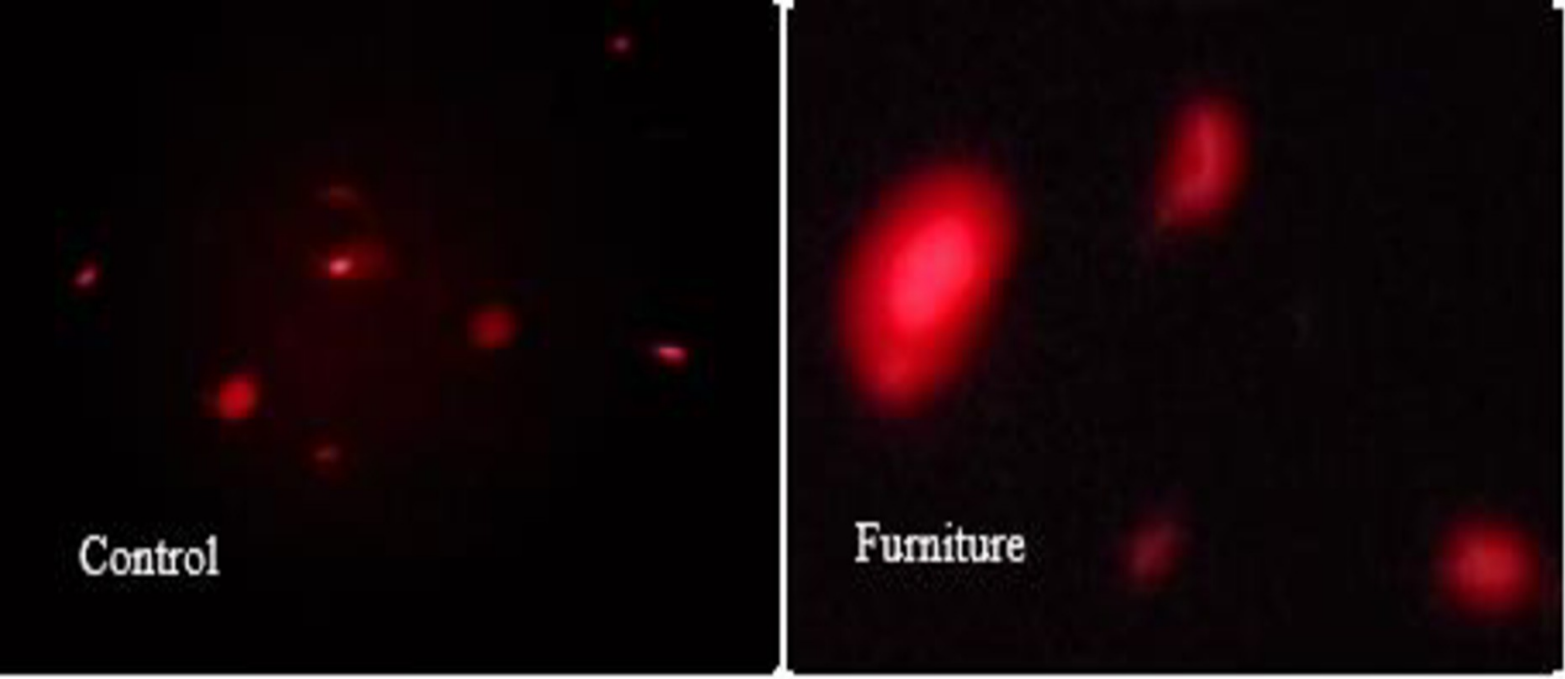
Fluorescence photomicrograph of blood lymphocytes from control and furniture group stained with Acridine Orange after processed through Single Cell Gel Electrophoresis (Comet assay).

In control group cells with intact DNA were more dominated and few cells showed short tailed comet. A significant DNA damage described by clearly visible comet tail was observed in all groups of occupationally exposed workers. Maximum DNA fragmentation was found in the blood cells of furniture workers compared to other industrial groups and controls (Fig 1).

### Comet assay parameters

To evaluate the index of DNA damage seven parameters of comet head and tail were compared between control and industrial group.

Comet head and length increased significantly (P<0.001) in the furniture and brick kiln group compared to the control. %DNA in comet head reduced significantly (P<0.001) in furniture group compared to control. A significant increase was observed in the tail length of the brick kiln (P<0.05) and furniture (P<0.001) group compared to the control. %DNA tail, tail moment, and OTM showed a significant increase in furniture (P<0.001) and welding (P<0.05) group compared to control (Table 2).

**Table 2:** Comparison of different comet parameters between control and different industries.

Comet Parameters	Groups					
	Control	Furniture	Brick kiln	Welding	Pesticide	Paint
**Comet length**	99.55±2.26	143.60±4.37***	122.00±5.95***	105.70±3.23	100.80±3.31	98.19±3.10
**Head length**	78.59±2.04	115.90±3.56***	98.25±4.85***	82.79±2.52	75.50±3.04	76.45±3.13
**%DNA head**	89.08±0.65	79.49±2.11***	86.15±1.52	84.12±1.63	89.95±1.03	87.00±2.39
**Tail length**	16.92±0.78	32.07±2.02***	23.85±2.24*	22.25±1.63	11.31±0.94	11.74±1.33
**%DNA tail**	10.20±0.56	21.15±2.12***	15.13±1.60	17.57±1.73**	10.05±1.02	15.29±2.68
**Tail moment**	2.29±0.19	6.60±0.70***	4.09±0.76	5.61±1.01**	1.71±0.19	2.08±0.47
**Olive tail moment**	3.49±0.28	7.71±0.66***	4.63±0.60	5.61±0.64*	2.22±0.23	2.94±0.47

Values expressed as Mean±SEM.

*P<0.05

**P<0.01

***P<0.001.

**Fig 2:**
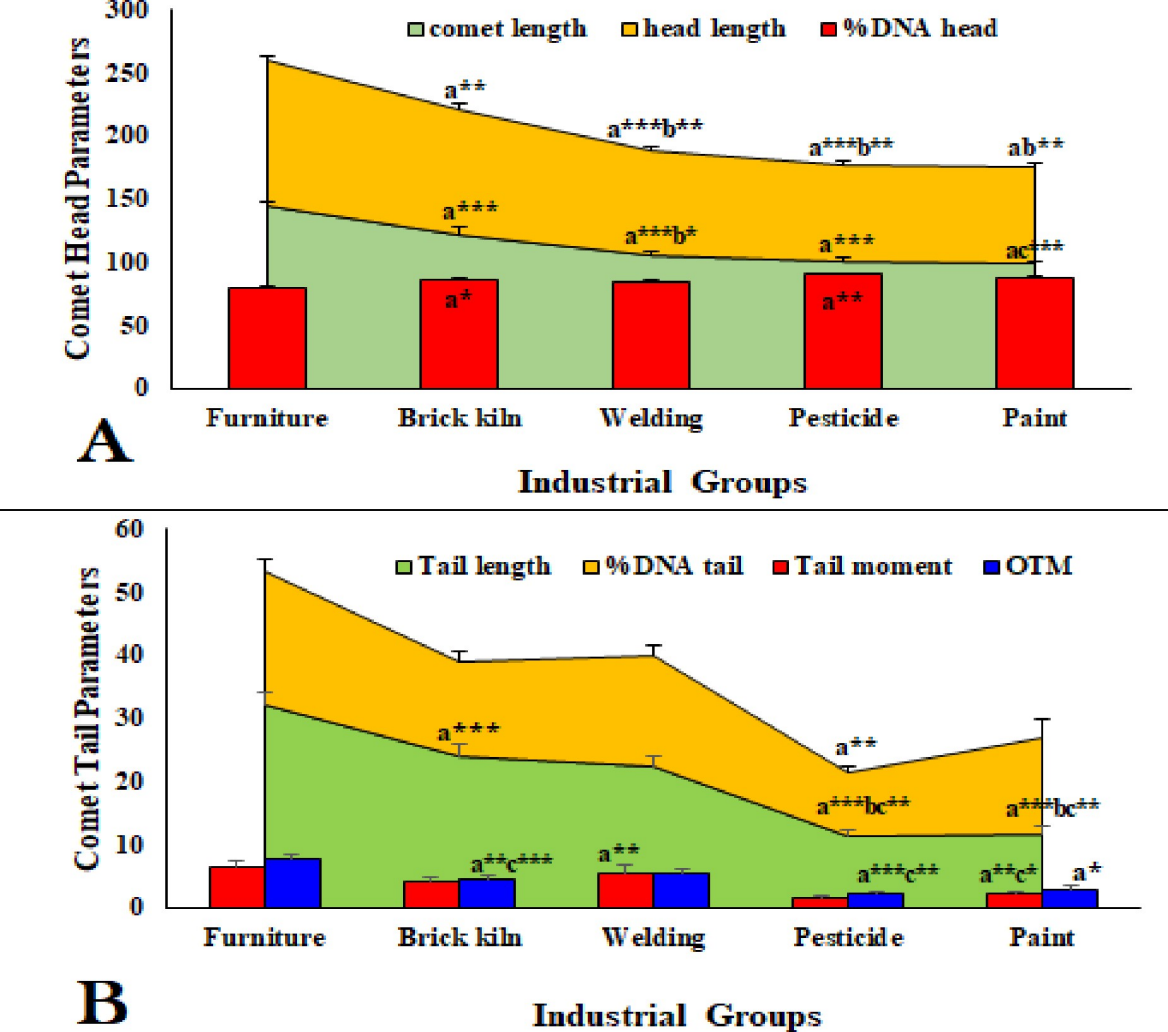
Different comet parameters associated with (A) comet head and (B) comet tail in lymphocytes of industrial workers. a = Furniture VS other industries, b = Brick kiln VS other industries, c = Welding VS other industries. *P<0.05, **P<0.01, ***P<0.001.

**Fig 3:**
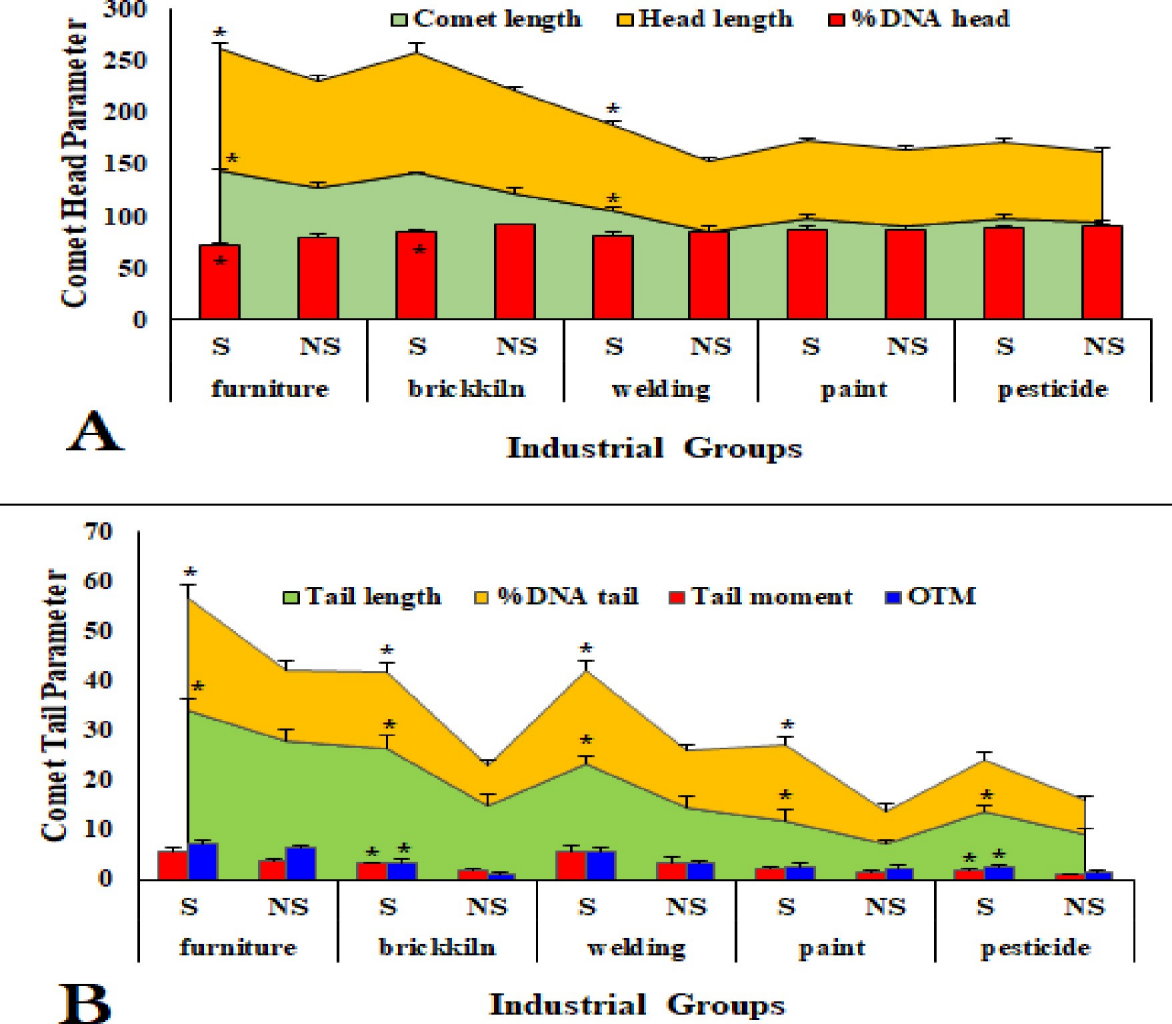
Different comet parameters associated with comet head and tail measured by comet assay in smokers (S) and non-smokers (NS) of exposed workers from different industries. *P<0.05, **P<0.01, ***P<0.001.

Index of DNA damage was significantly dominated in furniture group compared to other industries. Although brick kiln and welding group showed prominent differences in comet length, %DNA head, tail length, %DNA tail, tail moment, and OTM compared to furniture group (Fig 2A and 2B). The degree of DNA damage was increased significantly in smokers (S) of each industrial group compared to their respective non-smokers (NS) (Fig 3A and 3B).

### *OGG1* gene

**Fig 4:**
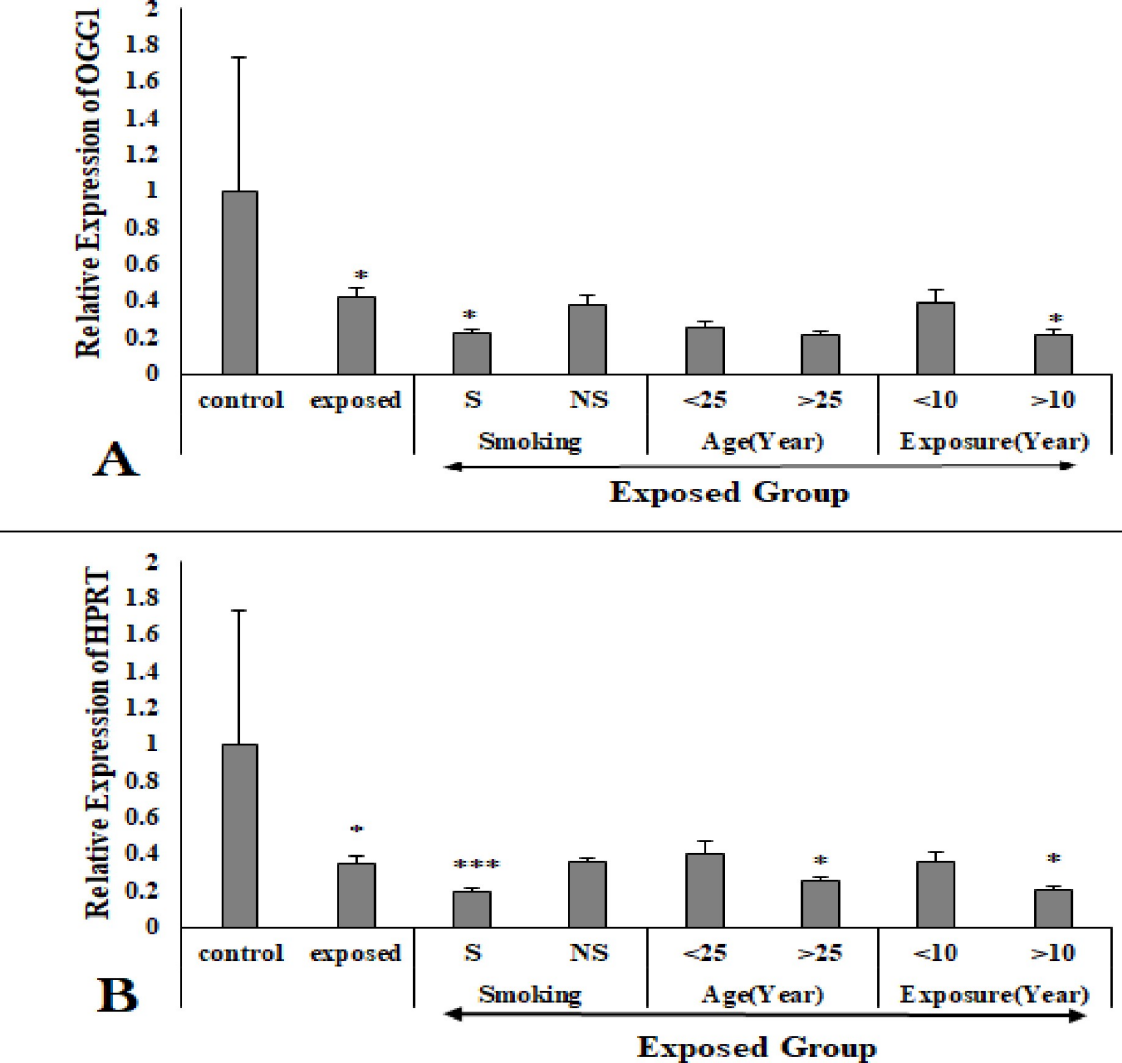
Relative expression of *OGG1* (A) and *HPRT* (B) gene in control and exposed workers. S = smoker, NS = non-smokers. *P<0.05, **P<0.01, ***P<0.001.

Expression of OGG1 was significantly (P<0.05) downregulated in exposed group compared to the control group. Reduced expression of *OGG1* gene was observed in smokers (P<0.05) of exposed group compared to their respective non-smokers. Similar trend was noticed in workers with >10 year of occupational exposure (P<0.05) compared to workers with <10 years exposure. Decreased expression of OGG1 gene was observed in workers with >25years of age compared to workers with <25 years of age, although the difference was statistically non-significant (Fig 4A).

### *HPRT* gene

Expression of *HPRT* gene significantly (P<0.05) downregulated in exposed group compared to the control. *HPRT* gene expression was decreased significantly (P<0.001) in exposed smokers group compared to exposed non-smoker group. Reduced HPRT expression was observed (P<0.05) in workers with >25 years of age compared to workers with <25 years of age. Similar decrease (P<0.05) was observed in workers with >10year exposure compared to workers with <10 years exposure (Fig 4B).

### Co-expression analysis of selected genes

**Fig 5:**
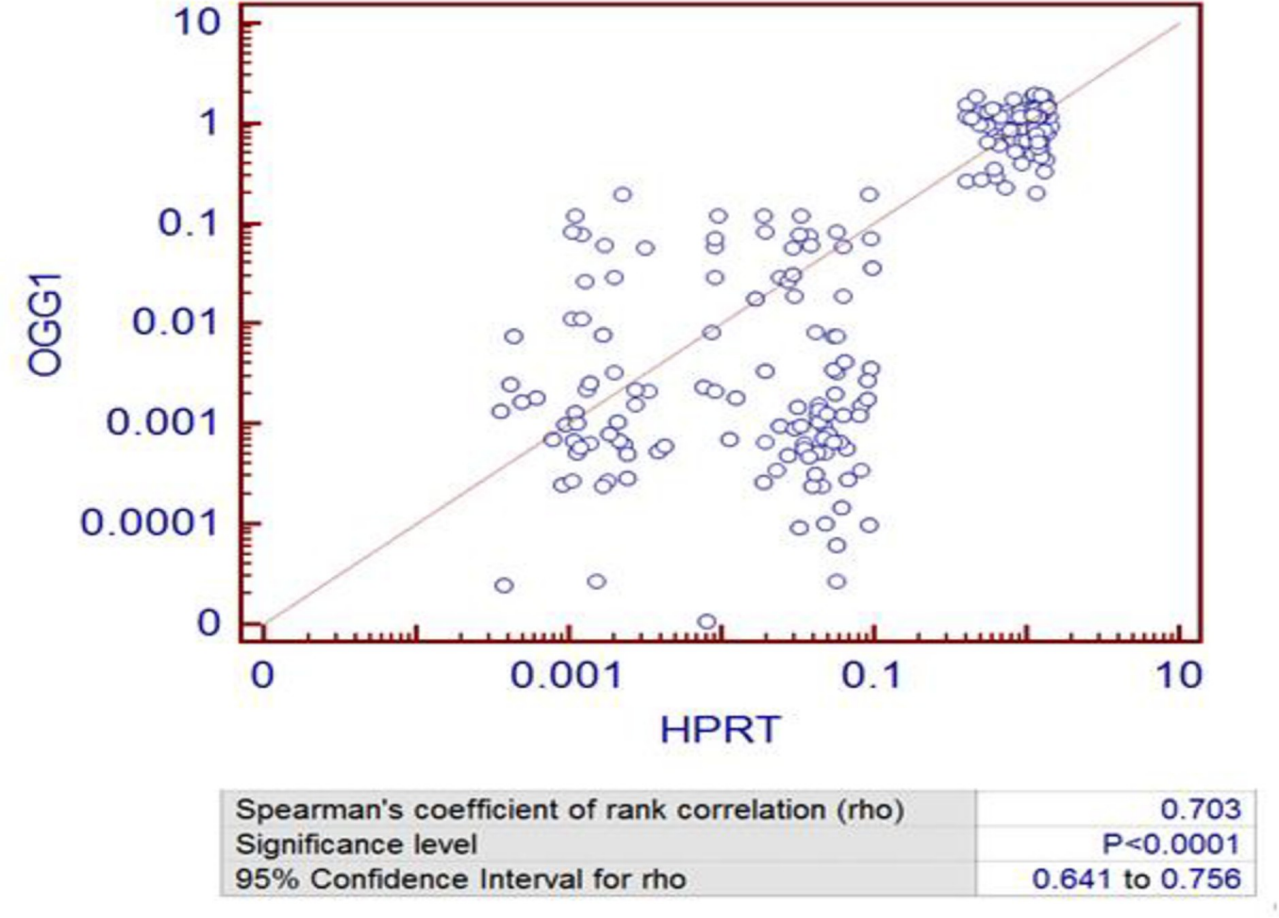
Co-expression analysis of *OGG1* gene vs *HRPT* gene in industrial exposed workers.

Expression level of OGG1 and HPRT was correlated using the co-expression analysis (Fig 5). Co-expression analysis showed a significant positive correlation between the expression levels of *OGG1* gene and *HPRT* gene (rho = 0.73; p<0.0001) in industrial workers.

### Metal estimation

**Fig 6:**
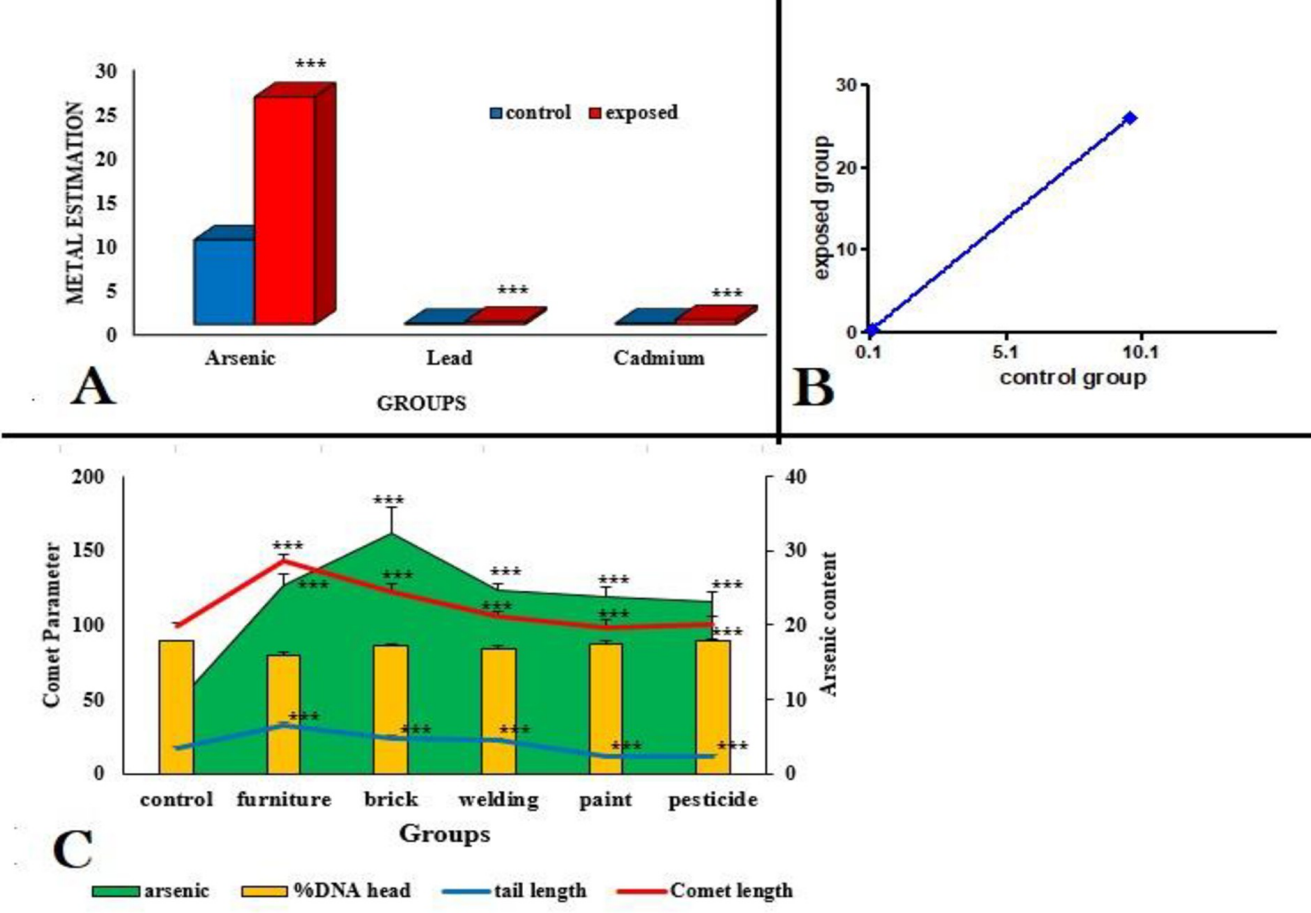
Deposition of metal content (arsenic, lead, cadmium) in blood samples of occupationally exposed workers of different industries (A). Calculated regression line showing metal deposition in control and exposed workers ((b = 2.718 ± 0.01367; F _(1,2)_ = 39521; P = 0.0032) (B). Association of arsenic content with different parameters of comet assay (C). ***P<0.001.

Arsenic, cadmium and lead content was significantly (P<0.001) higher in exposed group compared to control group. Blood arsenic levels were significantly (P<0.001) higher in exposed group compared to blood cadmium and lead levels in same group (Fig 6A). Regression analyses of variance showed a significant increase in metal deposition (b = 2.718 ± 0.01367; F _(1,2)_ = 39521; P = 0.0032) in exposed group against metal deposition in unexposed control group (Fig 6B). Within industrial group, arsenic deposition was significantly increased (P<0.01) in brick kiln and furniture group compared to other industries (Table 3).

**Table 3:** Arsenic content in blood samples of controls and workers of different industries.

Groups	Blood Arsenic content (μg/L)
Control	9.67±1.03
Exposed	25.80±0.50***
Brick kiln industry	32.42±3.42^a^***
Furniture industry	25.32±01.48^a^***^b^*
Pesticide industry	24.72±0.81^a^***^b^**
Paint industry	23.90±1.18^a^***^b^**
Welding industry	23.15±1.36^a^***^b^***

Values expressed as Mean±SEM.

a = control versus industries groups

b = brick kiln industry versus other industries groups.

*P<0.05

**P<0.01

***P<0.001.

### Association of arsenic content with comet parameters

Correlation of comet parameters was analyzed with total arsenic content in blood samples of exposed workers from targeted industries. Significant (P<0.001) correlation was observed in comet length, tail length and %DNA head in furniture, brick kiln, paint, pesticide and welding group against total arsenic content in blood (Fig 6C).

## Discussion

Worldwide millions of people are occupationally exposed to various toxic agents at their workplaces [31]. Therefore, it is important to quantify the risk assessment in biological samples of occupationally exposed workers. In this study DNA damage was detected using comet assay, relative expression of OGG1 and HPRT gene was analyzed and their association was established with different demographic parameters. Various mechanisms tangled with arsenic induced genotoxicity including DNA repair inhibition, oxidative stress, DNA damage, and mutagenesis [32]. Chronic exposure to arsenic contaminated drinking water is related to an increased risk of lung, bladder, kidney, and skin cancer [33,34]. Likewise, evidence of tumors formation was reported in mice chronically exposed to arsenic [35]. In the present study, the index of DNA damage was measured using different parameters of the comet assay. The extent of DNA fragmentation and deposition of arsenic content was higher in industrial workers compared to control. Furniture, brick kiln, and welding group showed prominent DNA damage compared to paint and pesticide group. Our group had reported significant deposition of arsenic in water, rice, fruit, and vegetable collected from industrial areas [36]. The frequency of DNA damage was higher in lymphocytes of children living in the vicinity of mining sites contaminated with lead and arsenic compared to those living away [35]. Increased tail length was observed in glassworkers chronically exposed to arsenic [37]. Polish copper smelters have a significant increase in tail moment compared to the control subjects [38]. Arsenic is used either as an ingredient or released as a byproduct in five industries of our concern. Our results revealed a positive association between arsenic exposure and the magnitude of DNA fragmentation in industrial workers. Limited data have been published so far to enumerate the DNA health of industrial workers. Interestingly our study is one of its own type of multiple comparison-based studies, which quantifies the genetic damage using seven parameters of comet assay and their comparison not only with controls but between workers of our focus industries. This would be helpful to identify the industry majorly involved in the induction of genetic instability. The furniture industry has been listed as the top contributor of DNA damage. Arsenic has long history in wood industry. Complex arsenic compounds are used for preservation of wood and attack from termite, fungi and other pests which threaten the integrity of wood. Lead arsenate is used as insecticide and wood preservative, copper chromated arsenate (CCA) to prevent wood from fungal and bacterial attack. Ammonical copper zinc arsenate is waterborne preservative used to retain quality of timber. After furniture industry, genetic damage was evident in the brick kiln and welding industry compared to others. In Pakistan, coal and wood are the major fuel in brick kiln industry generating harmful effects in environment and put the health of workers at great risk. Most of wood and coal used in making of bricks are large reservoir of arsenic. A significant increase in the tail moment [17], comet tail length [4,39], %DNA tail was observed in welders of different areas [16].

Long term heavy metals exposure is highly threatening to DNA health and may affect the expression of various DNA repair genes. Downregulation in the *OGG1* gene and its enzymatic activity has been commonly observed upon heavy metal exposure like arsenic, cadmium, and chromium [40–42]. *OGG1* encodes an enzyme involved in the removal of DNA lesion 8-OxoG. 8-OxoG is widely accepted as an oxidative DNA damage biomarker and arsenic exposure induces 8-OxoG generation [43]. *OGG1* knockout mice showed significant accumulation of DNA lesion 8-OxoG upon arsenic exposure [44]. Our results revealed downregulation of *OGG1* expression in arsenic exposed industrial workers compared to controls. Increased oxidative DNA damage was reported in nickel exposed workers with decreased *OGG1* expression [45]. Reduced activity of OGG1 suggested the limited ability of repair mechanism to remove DNA lesions and will increase the risk of DNA damage ultimately leading to carcinogenesis. A high frequency of 8-OxoG was observed in patients with arsenic-induced skin cancer [46].

Hypoxanthin guanine phosphoribosyl transferase control DNA damage by recycling nucleotides *HPRT* gene acts as a part of DNA repair mechanisms, functions in the protein salvage pathway using degraded DNA and RNA products and transforming them into purines. Expression of *HPRT* was significantly down-regulated in industrial workers of our study compared to controls. *HPRT* knockdown results in deregulation of genes essential in RNA metabolism, DNA replication, and repair [47]. ROS production due to arsenic exposure causes oxidative damage which induces aberration in HPRT resulting in loss of ATP and cell death. Moreover, mitochondrial depletion occurs due to arsenic induced DNA damage leading to decreased ATP production and cell death (Singh et al., 2011). Arsenic has the ability to mimic the phosphate group of ATP, forming ADP-arsenate, interrupting glucose metabolism and loss of energy bonds [48]. Possibly altered levels of HPRT may inhibit the protein salvage pathway to reuse degraded DNA/ RNA to form purine thus affecting the ability of the DNA repair mechanism.

The industrial group was further categorized as smokers and non-smokers. Significant differences in various comet parameters and expression deregulation of *OGG1* and HPRT gene between smokers and non-smokers was a clear indication of genetic instability or DNA damage. About 4500 toxic chemicals are present in cigarette smoke which introduced oxidative stress, genetic aberrations, mutation, and DNA strand breaks [49]. Cigarette smoke and arsenic act synergistically as a major disaster of health, either inducing oxidative stress or restricted antioxidants defense system resulting in DNA damage [50]. Our group had reported depleted activity of antioxidant enzymes like CAT, SOD, glutathione peroxidase in industrial workers [51]. One cause of bladder cancer is smoking along with arsenic exposure [48]. Chronic exposure to arsenic leads to its accumulation in the body which interferes with different enzymatic activity and cellular processes [51]. Arsenic is known to deactivate the function of more than 200 enzymes [52]. Present and previous data suggested that arsenic exposure act synergistically with smoking alter *OGG1* and HPRT reliability which makes it unable to repair the DNA lesion, ultimately will affect the genetic stability and DNA integrity of occupationally exposed workers hence, putting the workers at high risk of health issues.

Age is a critical factor because it would decrease the body’s ability to minimize the damage. Upon arsenic exposure, aged mice were more vulnerable to toxicity compared to young and middle age mice [53]. Prolonged arsenic exposure either due to age or occupational is a contributing element to initiating different diseases. Our results revealed reduced expression of *OGG1* and *HPRT* in workers >25 years of age compared to workers with <25 years of age. Same is observed for workers with >10-year exposure compared to workers with <10-year exposure. Methylation capacity decreases with age which leads to the accumulation of arsenic inside the body resulting to putting DNA integrity at risk [54]. Possibly aged workers with prolonged exposure may have lacked the efficient removal of arsenic in their urine due to slow body response to arsenic accumulation.

### Conclusion

The current study concluded that industrial workers occupationally exposed to arsenic showed an increased index of DNA damage and variation in *HPRT* and *OGG1* gene expression. It has been suggested that industrial workers are at great risk of genetic instability due to oxidative damage/stress induced by down-regulation of *OGG1* and HPRT gene. This would affect the purine recycling mechanism which in turn disturbed the DNA repair pathway. Furthermore, prolonged arsenic exposure, increasing age, and smoking habits act synergistically to make the workers’ health at great risk and make them more vulnerable to genetic damage. Personal protective care should be taken in the occupational area, and there is a need for proper awareness about toxicants and safety limits.

Blood arsenic content in occupationally exposed workers was elevated compared to lead and cadmium content. Therefore, genetic damage and instability possibly attributed toward arsenic toxicity compared to other metals estimated in present study. The main limitations of current study are (i) sample collection from focused area of Central and South Punjab. (ii) estimation of only three targeted metals. (iii) limited number of industries. (iv) estimation of metal content only in blood samples. Therefore, if the sampling area has expanded, number of targeted industries would increase and multiple metals would be measured in blood samples of industrial workers, this would generate more clear picture for metal induced genotoxicity in exposed workers.

## Methods

The experimental protocol for the use of Human was approved (CIIT/BIO/ERB/16/09) by the ethical review board COMSATS University Islamabad, Pakistan.

### Sampling

Blood samples of 300 occupational workers (n = 60/industry) were collected from the welding, brick kiln, furniture, pesticide, and paint industry of Central and South Punjab, Pakistan, along with age and gender matched 300 unexposed control subjects. Sample size was calculated using Sample Size Calculator (calculator.net) with 95% confidence interval and 5% margin of error. Inclusion criteria for industrial workers was minimum five-year exposure to respective industry. Whereas controls were unexposed healthy individuals with no history of any specific disease. All smokers of present study had current smoking status with consumption of at least 10–12 cigarette per day. Blood samples of control and exposed workers were collected in EDTA coated vacutainers. Lymphocytes were isolated from whole blood and processed for estimation of DNA damage using the comet assay protocol of Akram et al (2019) [55]. This observation was done at COMSATS University Islamabad, Pakistan. Verbal consent was taken from every participant. The experiments were completed in agreement with the principles of the Declaration of Helsinki [56].

### RNA extraction and cDNA preparation

RNA was extracted from whole blood using the Trizol reagent method [35]. Extracted RNA was quantified on a Nanodrop spectrophotometer (ND-100, USA).

### Expression analysis

Primers of OGG1, HPRT and β-actin were designed using IDT (Integrated DNA Technology), and coding sequences were obtained from ensemble genome browser. The primer sequences are given in Table 4.

**Table 4:** Primer sequence of *HPRT*, *OGG1* and *β-actin* gene.

Gene	Primer	Sequence
*HPRT*	Forward	5΄CTGAACGTCTTGCTCGAGAT3΄
*HPRT*	Reverse	5΄CCAGCAGGTCAGCAAAGGAA 3
*OGG1*	Forward	΄TCAGGAAAGCCGGAGAATTG3΄
*OGG1*	Reverse	΄CCCACACGGTGCTGTTTA3΄
*β-actin*	Forward	5΄TTCTCTGACCTGAGTCTCCTT3΄
*β-actin*	Reverse	5΄ACACCCACAACACTGTCTTAG3΄

A quantitative Real-Time PCR System (Applied Biosystem) was used to perform the qPCR reactions. The comparative mRNA expression of *OGG1*, *HPRT*, and β-actin was evaluated using 2^-delta delta CT^ analysis method.

### Wet acid digestion

Total arsenic, cadmium and lead content was measured in blood samples of control and industrial workers using the wet acid digestion method described by Yahaya et al (2013) [57]. Whole blood was heated in a mixture of nitric acid and hydrogen peroxide, until clear transparent solution was attained. Metals were estimated using flame atomic absorption spectrophotometer (Varian (AA240FS) at wavelength 193.7nm, slit (nm) 0.5, relative sensitivity 1, relative intensity 50, and current was 9mA.

### Quality assurance and quality control

In present study, for prevention of contamination of procedure, all glassware, pipette tips and stoppers were washed with 10% nitric acid and rinsed in distilled ionized water before they were used in procedure. For quality assurance of analytical method, blank and triplicate samples were analyzed and this quality assurance was further confirmed using water sample (unexposed area and measured for arsenic content, 0.0086μg/L). Limit of detection (LOD) for arsenic is 9.6 μg/L and limit of quantification (LOQ) for arsenic is 58.1 μg/L.

### Statistical analysis

Differences between industrial and control groups were analyzed using One-Way ANOVA, Tukey’s test, student’s t-test, X^2^ test and Spearman correlation analysis. Comparison was made between different industrial groups using One-Way ANOVA following post ANOVA multiple comparison Tukey’s test. Levels of selected metals were compared in control and exposed individuals using student’s t-test. Association of different demographic parameters like age, exposure time and smoking status was analyzed using X^2^ test. Spearman correlation analysis was used to correlate the expression value of selected genes. The whole data was analyzed using GraphPad Prism8.
